# Ag Nanoparticles Stabilized on Cyclodextrin Polymer Decorated with Multi-Nitrogen Atom Containing Polymer: An Efficient Catalyst for the Synthesis of Xanthenes

**DOI:** 10.3390/molecules25020241

**Published:** 2020-01-07

**Authors:** Samahe Sadjadi, Fatemeh Ghoreyshi Kahangi, Masoumeh Dorraj, Majid M. Heravi

**Affiliations:** 1 Gas Conversion Department, Faculty of Petrochemicals, Iran Polymer and Petrochemicals Institute, P.O. Box 14975112, Tehran 1497713115, Iran; masidor20@gmail.com; 2 Department of Chemistry, University Campus 2, University of Guilan, Rasht 4199613776, Iran; nf_ghoreyshi@yahoo.com; 3 Department of Chemistry, School of Science, Alzahra University, P.O. Box 1993891176, Vanak, Tehran 1993891176, Iran

**Keywords:** cyclodextrin polymer, catalyst, silver nanoparticles, xanthenes

## Abstract

In attempt to broaden the use of cyclodextrin polymer for catalytic purposes, a novel covalent hybrid system was prepared through growth of multi-nitrogen atom containing polymer (PMelamine) derived from reaction of ethylenediamine and 2,4,6-trichloro-1,3,5-triazine on the functionalized cyclodextrin polymer (CDNS). The resulting hybrid system was then utilized as a catalyst support for the immobilization of silver nanoparticles through using *Cuscuta epithymum* extract as a naturally-derived reducing agent. The catalytic activity of the catalyst, Ag@CDNS-N/PMelamine, for the synthesis of xanthenes through reaction of aldehydes and dimedone in aqueous media was examined. The results showed high catalytic activity and recyclability of the catalyst. It was believed that cyclodextrin in the backbone of the catalyst could act both as a capping agent for Ag nanoparticles and phase transfer agent to bring the hydrophobic substrates in the vicinity of the catalytic active sites and accelerate the reaction rate. Multi-nitrogen atoms on the polymer, on the other hand, could improve the Ag NPs anchoring and suppress their leaching.

Academic Editors: Paolo Lo Meo and Anna Trzeciak

## 1. Introduction

Within the past decade, noble metal nanoparticles (NPs) with narrow particle size distributions have received significant attention in various applications, such as catalysis. Metal NPs are known to provide high surface area of catalytically active sites [1,2,3,4]. However, the major problem for the noble metal NPs is their high tendency to form aggregate, thereby leading to loss of their main characteristics.

A possible way to address this issue is to stabilize the NPs through immobilizing them on an appropriate solid substrate such as inorganic oxides, carbon-based nanomaterials or insoluble polymers, or using soluble capping agents such as surfactants, ligands, or polymers [5,6,7,8,9].

One of the most known oligosaccharides for the catalytic purposes is cyclodextrin (CD). CD is a cyclic oligosaccharide with an exceptional cone-shape structure with hydrophobic exterior surface and hydrophilic interior space [10,11,12]. This feature allows CD to form an inclusion complex with hydrophobic reagents. As the outer surface of CD is hydrophilic, the hosted guest can be easily transferred to aqueous media. In this way, CD can act as a molecular shuttle. On the other hand, CD can serve as a stabilizing agent for nanoparticles and prevent their aggregation through efficient capping [13,14].

It is worth to mention that the activity of heterogeneous catalysts strongly depends on the loading amount of their immobilized homogenous moieties [15]. An innovative way for increasing the loading amounts of NPs is immobilization of metal ions onto cross-linked polymeric networks [15,16]. These polymeric networks have high porosity, high surface area, and large amounts of attaching sites to grab metal ions [17]. Polymeric networks have many coordination sites, which can adsorb the large amounts of metal ions. Besides, they are more thermally and chemically stable than conventional supports [8].

CD-based polymers (CDNSs) can be prepared through reaction of CDs with a cross-linking agent. This class of compounds benefits from the features of CD and the polymeric network. Nanocomposites of CDNs and metal nanoparticles are also very interesting materials. In these systems, CDs can stabilize nanoparticles and improve catalytic properties [18].

Among the various noble metal NPs, silver nanoparticles (Ag-NPs) have received particular attention in view of their unique applications and characteristics. In recent years, Ag-NPs witnessed growing applications for catalysis, sensing, electronic, biological labeling [19], drug delivery [20], water treatment [21], etc.. Mainly, the Ag-NP preparation method involves reduction of silver ions in the solution or in gaseous environments [22]. Use of chemical reducing reagents is not environmentally benign [22,23]. Alternatively, naturally derived reducing agents can be used for the synthesis of nanoparticles [16,17,18,19]. Use of this class of reagents benefits from some advantages such as low cost and non-toxicity. Moreover, the reduction process mostly can be carried out in aqueous solution in a single-step procedure. Nowadays, using plant extracts as reducing and stabilizing agents for the synthesis of metallic nanoparticles is considered to be an eco-friendly and rapid strategy.

Xanthene derivatives are key biologically active chemicals with diverse important pharmacological properties such as antibacterial, antiviral, and anti-inflammatory [24,25]. These compounds are found in the structure of drugs used in photodynamic therapy [26,27,28]. Moreover, xanthenes can be applied for the development of pH-sensitive fluorescent materials and dyes. Considering high utility of xanthenes, many researchers devoted their research to develop efficient procedures for the synthesis of these chemicals [29,30]. As examples, some catalysts such as ionic liquid [31], TiO_2_-SO_3_H [32], and acid functionalized SiO_2_ [33,34,35] have been reported for xanthene synthesis.

In our following research on CD-based heterogeneous catalysts [36,37,38,39,40], herein we wish to report a novel catalyst support based on growth of multi-nitrogen-containing polymer on the functionalized CDNS. More precisely, amine functionalized CDNS was prepared and reacted with ethylenediamine and 2,4,6-trichloro-1,3,5-triazine under basic condition to allow multi-nitrogen atom containing polymer growth. Then, the hybrid system was applied for the immobilization of silver nanoparticles through reduction with *Cuscuta epithymum* extract as a naturally-derived reducing agent. The reasons for use of this extract was as follow: availability in large quantity in our local area, very low cost, non-toxicity, and environmentally benign nature. The final hybrid system was then applied as a heterogeneous catalyst for promoting the synthesis of xanthene derivatives from reaction of aldehydes and dimedone in aqueous media. The generality of the developed protocol and the recyclability of the catalyst were also studied. Furthermore, to elucidate the roles of multi-nitrogen containing polymer and CDNS in the catalysis and disclose the merit of this catalyst, the catalytic activity of the catalyst was compared with Ag@CDNS, Ag@multi-nitrogen atom-containing polymer and some previously reported catalysts.

## 2. Result and Discussion

### 2.1. Characterization of Ag@CDNS-N/PMelamine

The morphology of CDNS was studied by recording its FESEM images. The images showed that bare CDNS showed plate-like morphology. Moreover, the EDS and elemental mapping analysis also confirmed its formation (Supplementary Materials Figure S1). Then, to study the effect of incorporation nitrogen functionality by treating with APTES on the morphology of CDNS, the FESEM image of CDNS-N was recorded. Again, the EDS and mapping analysis confirmed the formation of this compound (Figure S2). It was found that introduction of APTES can alter the morphology of the catalyst and lead to the more compact morphology. Moreover, the elemental mapping analysis showed almost uniform distribution of N atoms, confirming that functionalization was achieved throughout the CDNS uniformly. Next, the morphology of CDNS-N/PMelamine was studied. The FESEM image of this sample (Figure S3) was distinguished from CDNS and CDNS-N and showed aggregated-like morphology.

**Figure 1:**
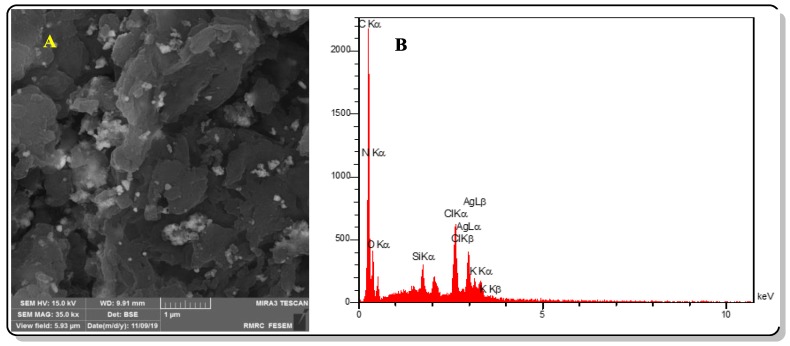
(**A**) FESEM image and (**B**) EDX analysis of the catalyst.

The FESEM images of Ag@CDNS-N/PMelamine catalyst is depicted in Figure 1A. As shown, the catalyst showed aggregate-like morphology that was different from CDNS, CDNS-N, and CDNS-N/PMelamine. The EDS analysis of the catalyst (Figure 1B) showed the presence of Si and O atoms, which are mainly representative of APTES. Moreover, the presence of Ag atoms can confirm the incorporation of Ag species in the hybrid catalyst. Also, the observation of C and N atoms can be attributed to the presence of CDNS-N/PMelamine. Notably, the observation of Cl atoms showed that in the course of condensation polymerization, some Cl atoms did not participate in the polymerization process. This can be due to the steric hindrance. In Table 1, the quantitative results of EDS analysis are summarized.

**Figure 2:**
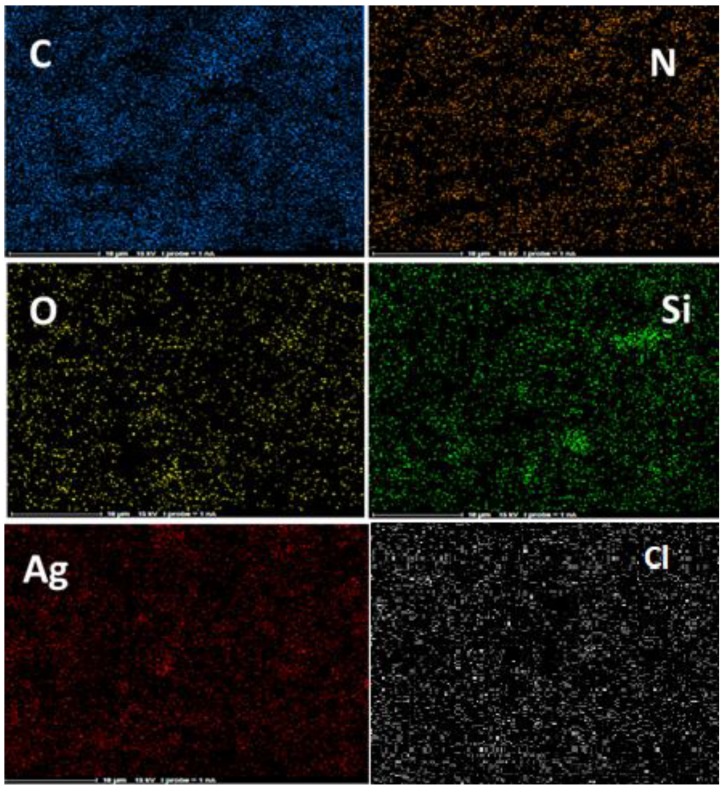
Elemental mapping of catalyst.

In the following, the elemental distribution in the Ag@CDNS-N/PMelamine catalyst was examined by elemental mapping analysis (Figure 2). As shown, Si atoms have been well distributed, confirming that CDNS has been uniformly functionalized with APTES. On the other hand, Ag distribution is also uniform. Similarly, this can be indicative of well dispersion of Ag on the support.

**Figure 3:**
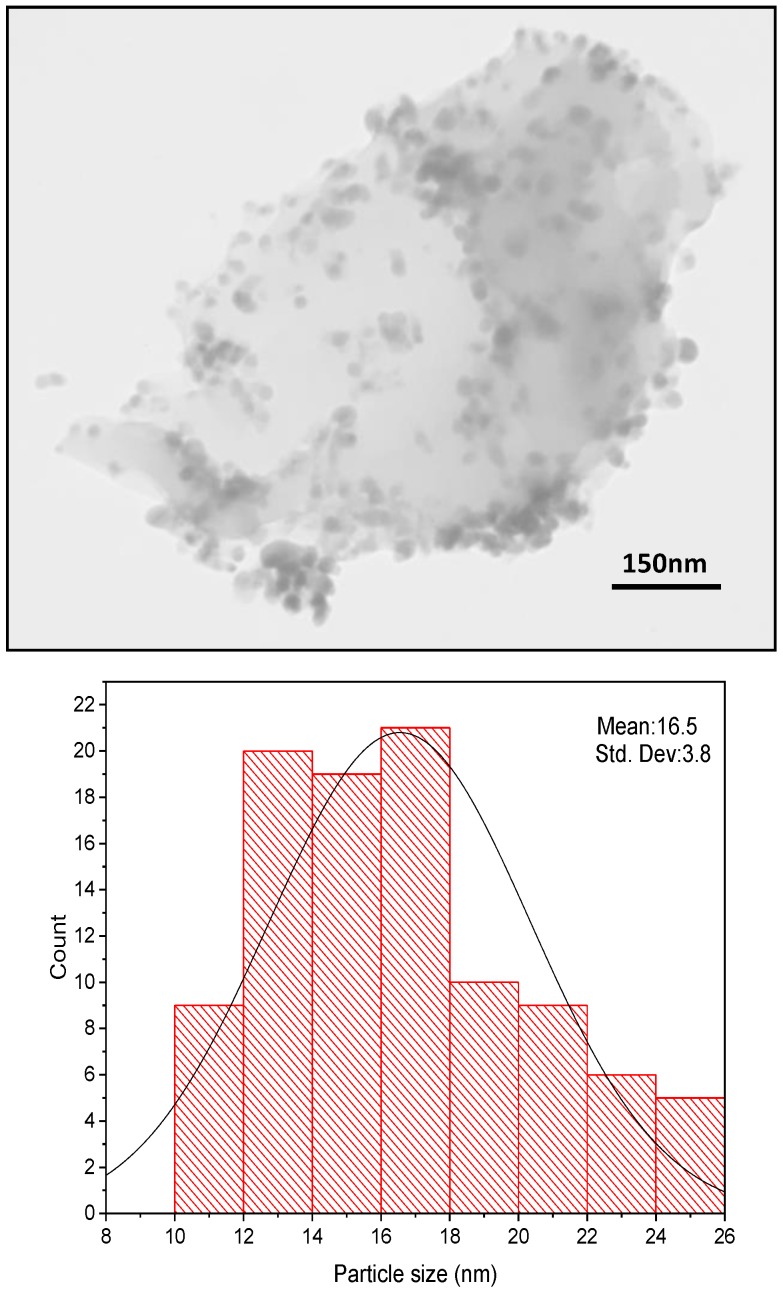
TEM image of catalyst.

Figure 3 shows the TEM image of Ag@CDNS-N/PMelamine. In the TEM image of the catalyst, the clear sheet-like structure of PMelamine could be observed. Moreover, the spherical dark spots in the photograph are representative of Ag NPs with average size of 16.5 nm ± 3.8. As shown in Figure 3, Ag NPs are dispersed on the support almost homogeneously and only on the edges of CDNS-N /PMelamine some aggregation is observed.

The silver content of Ag@CDNS-N/PMelamine was evaluated by using ICP-AES analysis. To this end, a known amount of Ag@CDNS-N/PMelamine was digested in a concentrated HCl and HNO_3_ solution. Subsequently, the resulting extract was analyzed applying ICP-AES. Using this approach, the content of Ag nanoparticles was measured to be 0.4 wt%.

**Figure 4:**
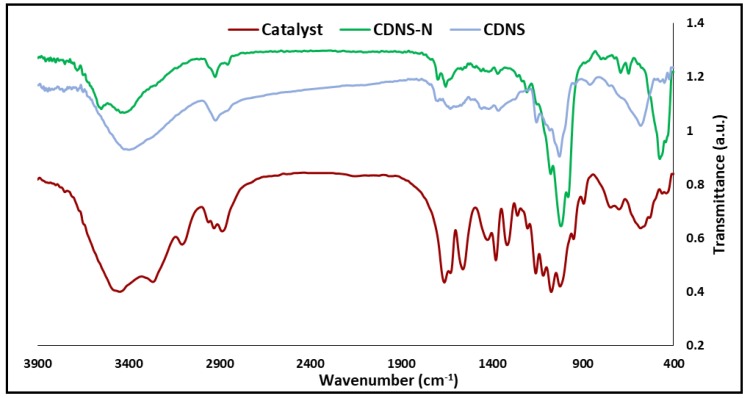
FTIR spectra of pristine CDNS, CDNS-N, and catalyst.

The FT-IR spectra of the CDNS, CDNS-N, and Ag@CDNS-N/PMelamine are exposed in Figure 4. The CDNS spectrum is in good agreement with previous reports [40] and showed the characteristic bands at 3400 cm^−1^ (-OH functionality), 2928 cm^−1^ (–CH_2_ groups), and 1700 cm^−1^ that can be assigned to the ester –C=O functionality, indicating successful cross-linking between the CDs and diphenyl carbonate. The FTIR spectrum of CDNS-N is very similar to that of CDNS. Noteworthy, the characteristic bands of APTES overlapped with those of CDNS. In the FTIR spectrum of Ag@CDNS-N/PMelamine, a strong band observed at 1664 cm^−1^ can be attributed to the vibration of the –C=N bonds that are present in the melamine rings. Furthermore, the bands at 2874 is indicative of –CH_2_ groups, while the bands at 3262 and 3400 cm^−1^ are representative of –NH and –OH functionalities, respectively.

**Figure 5:**
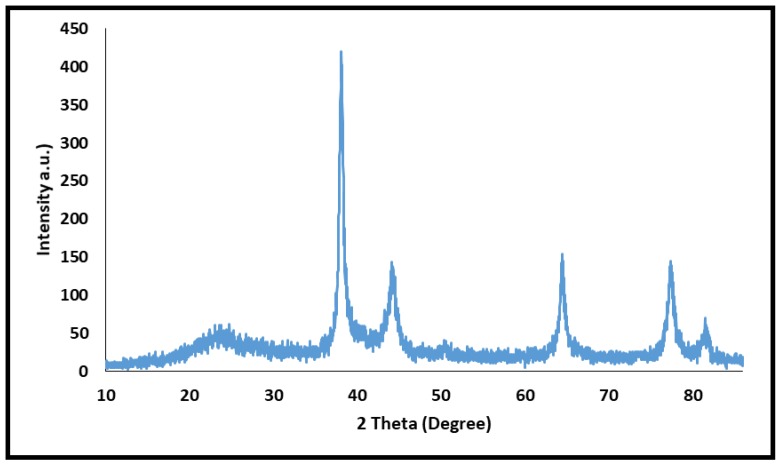
XRD pattern of the catalyst.

In the following, the XRD pattern of Ag@CDNS-N/PMelamine was recorded. As shown in Figure 5, the XRD pattern of the catalyst showed a broad halo at 2θ = 19–30° that can be assigned to the amorphous CDNS-N/PMelamine. This observation is in a good agreement with the previous reports, in which CDNS prepared via melting method showed amorphous structure [41]. According to the literature, the sharp bands at 2θ = 38.06°, 44.1°, 64.5°, 77.6°, and 81.5° can be attributed to the Ag(0) species [42], confirming the successful reduction of silver salt to the Ag(0) via *Cuscuta epithymum* extract.

Using BET, the specific surface area of Ag@CDNS-N/PMelamine was measured to be 19.4 m^2^ g^−1^. This value is higher than the specific surface area of bare CDNS (4 m^2^ g^−1^). This result confirmed that conjugation of PMelamine could improve the specific surface area of CDNS that is instinctively very low.

**Figure 6:**
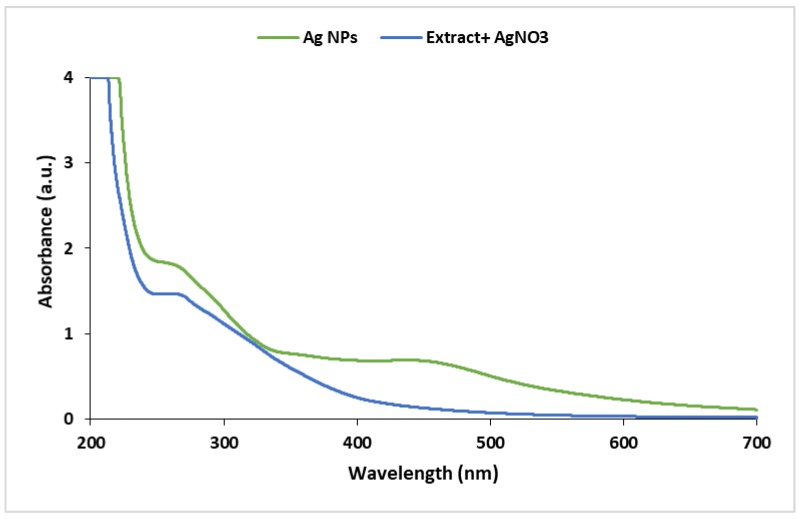
UV-visible spectrum of Ag nanoparticles, and the mixture of AgNO_3_ and plant extract.

The reduction of aqueous Ag^+^ ions to Ag^0^ by the Cuscuta leaf extract was confirmed by UV-visible spectroscopy. According to the literature [43,44], the characteristic band of Ag nanoparticles in the UV-visible spectrum occurs near λ_max_ = 430 nm. As depicted in Figure 6, in the UV spectrum of the mixture of the extract and AgNO_3,_ no band at λ_max_ = 430 nm was observed, while, after reduction, the Ag(0) band appeared in the UV-visible spectrum, confirming the successful reduction of Ag(I) to Ag(0).

### 2.2. Study of the Catalytic Performance

Confirming the formation of Ag@CDNS-N/PMelamine, its catalytic performance was scrutinized. Initially, two-component reaction of dimedone and benzaldehyde for the synthesis of xanthene was targeted as a model reaction. The reason behind this selection was the importance of xanthene derivatives as biologically active chemicals and their wide use for the synthesis of more complex chemicals. First, the reaction condition for the model reaction was optimized (Table 2). In this line, the model reaction was first performed in water as a solvent at ambient temperature in the presence of 0.02 mg Ag@CDNS-N/PMelamine. The result confirmed that under this condition, high yield of product (76%) was achieved. To increase the yield of the reaction, it was performed in various solvents with different polarities. The reason for use of water as a solvent was its environmentally benign nature. EtOH was also selected as a potential solvent. The reason for this choice was availability and non-hazardous nature of EtOH. Moreover, it was assumed that due to the different polarity, the solubility of the reagents can be improved in EtOH compared to pure water. The mixture of H_2_O:EtOH was also examined to provide a more ecofriendly solvent with improved potential for dissolving the reagents. THF and CH_3_CN were selected as non-polar solvents. The reasons for their selection were their low boiling points and their capability to dissolve the reagents. As tabulated, the mixture of H_2_O:EtOH with ratio of 2:1 resulted in the best result. Hence, it was selected as the reaction solvent. Subsequently, the effect of the reaction temperature was studied by elevating the reaction temperature to 50 °C. The results confirmed that increase of the reaction temperature led to the improvement of the reaction yield. However, there was not a linear relationship between the reaction temperature and the reaction yield and by increasing the reaction temperature from 50 to 70 °C, no improvement was observed in the reaction yield.

Then, it was investigated whether the increase of the amount of the catalyst from 0.02 to 0.03 g could increase the reaction yield. Gratifyingly, use of higher amount of the catalyst led to the higher yield of the desired product. However, further increase of the catalyst content had no positive effect on the yield of the product. Considering all of the results, the optimum reaction condition was using 0.03 g of Ag@CDNS-N/PMelamine at 50 °C in the mixture of H_2_O:EtOH.

In the following, the role of PMelamine in the catalysis was elucidated (Table 3). To this purpose, Ag@CDNS was synthesized. First, the optimum reaction condition for this catalyst was obtained (use of 0.04 g of Ag@CDNS at 60 °C in the mixture of H_2_O:EtOH). Under the optimum reaction condition, the catalytic activity of Ag@CDNS was 78% which was lower than that of Ag@CDNS-N/PMelamine. Moreover, examining of the catalytic activity of Ag@CDNS for promoting the model reaction under optimum reaction condition found for the catalyst confirmed that under that optimum reaction condition, Ag@CDNS led to lower yield of the corresponding product (70%). Notably, examining the catalytic activity of Ag@CDNS-N confirmed that the catalytic activity of this control sample was similar to that of Ag@CDNS and much inferior compared to that of the main catalyst.

This observation indicated the contribution of PMelamine to the catalysis. It was postulated that the abundant nitrogen containing functionalities on PMelamine could enhance the anchoring of Ag NPs. To verify this assumption, the Ag contents for Ag@CDNS and Ag@CDNS-N/PMelamine were measured via ICP. The results showed that the Ag loading in Ag@CDNS-N/PMelamine (0.4 wt%) was higher than Ag@CDNS (0.27 wt%). This observation confirmed that PMelamine effectively contributed to the Ag stabilization.

Next, the contribution of CDNS in the catalysis was studied. Similarly, the optimum reaction condition for this catalyst was first found as use of 0.035 g of Ag@PMelamine at 50 °C in the mixture of H_2_O:EtOH. The catalytic activity of this control catalyst was 80%. The comparison of the catalytic activity of Ag@PMelamine with that of Ag@CDNS-N/PMelamine under the optimum reaction condition found for the catalyst also showed the inferior activity of Ag@PMelamine (72%) compared to Ag@CDNS-N/PMelamine. According to the literature [10,11], the role of CDNS in the catalysis can be assigned to the capability of CDs to encapsulate the hydrophobic substrates and formation of inclusion complex. This feature allows CDs to act as phase transfer agents. On the other hand, CD can act as a capping agent for Ag NPs [45,46].

Next, it was elucidated whether this protocol could be generalized to other aldehydes. In this regard, various aldehydes with different functional groups and electron densities were examined. The results (Table 4) confirmed that Ag@CDNS-N/PMelamine could promote the reaction of various aldehyde derivatives with electron donating or electron withdrawing groups to furnish the corresponding products in high yields. Studying the furfural as substrate, it was found that this protocol can be generalized to heterocyclic substrates.

In the following, to investigate the efficiency of Ag@CDNS-N/PMelamine, the reaction condition and the efficiency of the catalyst were compared with those of some of the previously reported catalysts. Notably, as the reaction condition for each reported catalyst is different, the comparison cannot be accurate and only provides an insight into the activity of the catalyst. The results, tabulated in Table 5, revealed that various metallic catalysts have been reported for this organic transformation. As shown, this model reaction has been performed both in solvent and under solvent-free condition. From the data in Table 5, it can be concluded that some of the metallic catalysts such as Nano-NiO and Nano-ZnO were not effective for this reaction. Comparing the reaction temperatures of the tabulated catalysts, it can be seen that Ag@CDNS-N/PMelamine could promote the reaction in lower reaction temperature to furnish the product in comparative yield. Regarding the reaction time, it can be seen that this reaction was reported both in very short and very long reaction time and the reaction time of that Ag@CDNS-N/PMelamine can be considered as a relatively short one. On the other hand, Ag@CDNS-N/PMelamine could catalyze the reaction in aqueous media that is environmentally benign solvent. Considering all of these results, it can be concluded that Ag@CDNS-N/PMelamine can be classified as an efficient catalyst.

### 2.3. Reaction Mechanism

**Figure 7:**
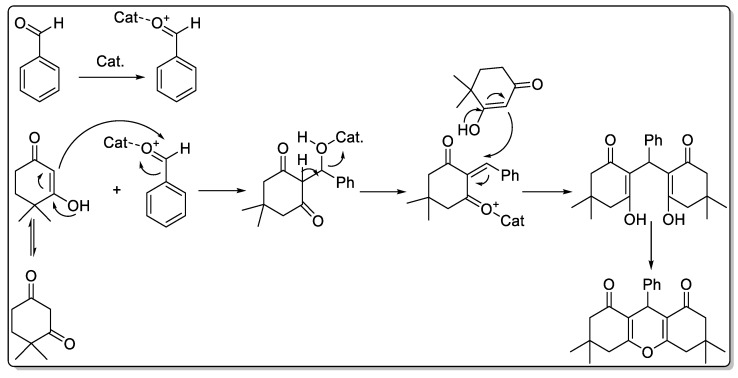
Plausible reaction mechanism for the synthesis of xanthenes.

Synthesis of xanthenes using silver nanoparticles has been previously reported [49]. According to the literature, the catalyst can activate aldehyde. On the other hand, CDNS in the structure of the catalyst can effectively act as a phase transfer agent and bring the hydrophobic substrates in the vicinity of the catalytic active sites. In the next step, the enole formed from dimedone reacted with the activated aldehyde to furnish an intermediate that then tolerates dehydration and reaction with second dimedone molecules. Finally, dehydration and cyclization leads to the formation of the desired product (Figure 7).

### 2.4. Catalyst Recyclability

**Figure 8:**
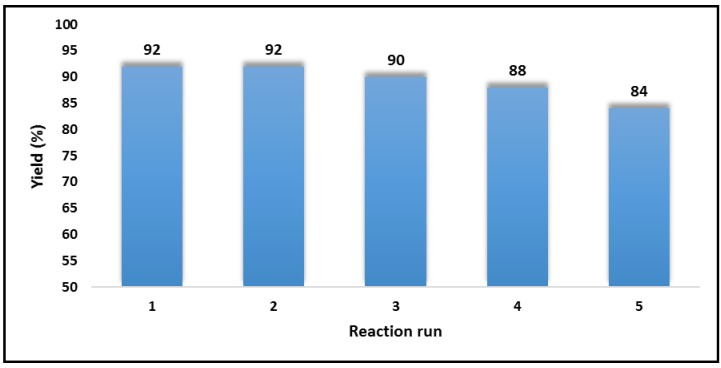
Recyclability of Ag@CDNS-N/PMelamine for the synthesis of model reaction.

The final feature of Ag@CDNS-N/PMelamine which was studied was its recyclability. This is an important characteristic of heterogeneous catalysts that renders them suitable for large scale use and industrialization. To this purpose, the recovered Ag@CDNS-N/PMelamine from model reaction was washed and dried and then applied as catalyst for the next run of the same reaction under similar reaction condition. This cycle was repeated for five reaction runs. In Figure 8, the yields of the desired product in the presence of fresh and recycled catalysts are summarized and compared. As shown, Ag@CDNS-N/PMelamine showed high recyclability and could be successfully recycled with slight loss of the catalytic activity.

**Figure 9:**
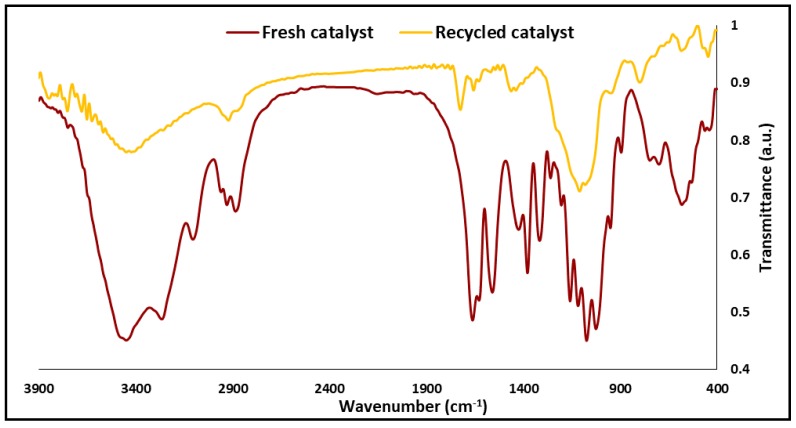
Comparison of FTIR spectrum of the recycled Ag@CDNS-N/PMelamine after five reaction runs with that of the fresh catalyst.

To further study the effect of recyclability on the catalyst, the recycled Ag@CDNS-N/PMelamine after five consecutive reaction runs was analyzed with ICP. Gratifyingly, the ICP results confirmed that recycling did not cause significant leach of Ag NPs and only slight loss of Ag NPs (3 wt% initial loading) was detected. On the other hand, the recycled catalyst (after five reaction runs) was also characterized via FTIR spectroscopy to elucidate whether recycling could destruct the structure of the catalyst. The FTIR spectrum of the recycled Ag@CDNS-N/PMelamine (Figure 9) was very similar to that of the fresh one, indicating that Ag@CDNS-N/PMelamine was stable under recycling.

**Figure 10:**
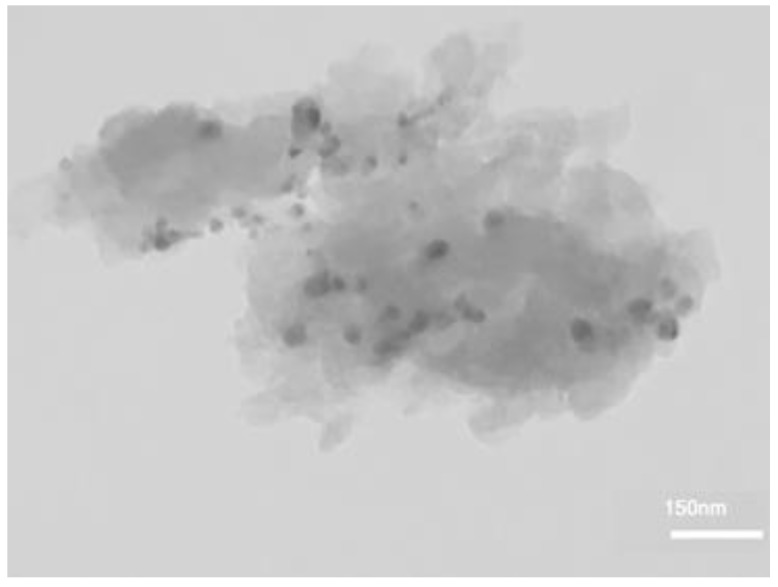
TEM image of the recycled catalyst after 5 reaction runs.

The morphology of the recycled catalyst after five reaction runs was also studied by recording its TEM image. As shown in Figure 10, the morphology of the recycled Ag@CDNS-N /PMelamine is similar to that of the fresh catalyst. The measurement of the Ag average particle size (16.8 nm ± 4.5) also confirmed that recycling did not induce significant aggregation.

## 3. Experimental

### 3.1. Materials and Instrumentation

All chemicals and reagents utilized for the synthesis of the catalyst and investigation of its catalytic activity, including 2,4,6-trichloro-1,3,5-triazine (TCT), ethylenediamine (EDA), (3-amino propyl) triethoxysilane (APTES), AgNO_3_, K_2_CO_3_, diphenyl carbonate, β-cyclodextrin, aldehydes, dimedone were purchased from Sigma-Aldrich and used as received without any further purification. To reduce Ag(I) to Ag(0), the extract of leaves of *Cuscuta epithymum* that were collected from Banaruiyeh District, in Larestan, Iran was used.

The synthesized hybrid catalyst was characterized using energy dispersive X-ray spectroscopy(EDS), FESEM, ICP, X-ray diffraction (XRD), Fourier transform infrared (FT-IR) spectroscopy, thermogravimetric analysis (TGA), transmission electron microscopy (TEM), and Brunauer-Emmett-Teller measurements (BET). FESEM and EDS analyses were done using a Bruker XFlash 6/100. XRD pattern of the as-synthesized sample was recorded from 2θ 8 to 90° on a (Siemens, model D5000, Karlsruhe, Germany), using Cu Kα radiation. TG analysis was performed with a (Mettler-Toledo, model Leicester, Leicester, UK) at a scanning rate of 10 °C min^−1^ from room temperature up to 800 °C under nitrogen flow. FT-IR spectra were undertaken with a PerkinElmer Spectrum 65 instrument. TEM analysis was performed using a Philips CM30 electron microscope operating at 300 kV. To perform this analysis, the samples were prepared by evaporating much diluted suspensions on carbon-coated cupper TEM grids. A BELSORP Mini II apparatus (BEL Japan, Inc., Osaka, Japan) was utilized to study the textural properties of the catalyst. To perform ICP analysis, ICP-AES Varian, Vista-pro (Salt Lake City, Australia) was used.

### 3.2. Synthesis of the Catalyst

#### 3.2.1. Synthesis of CDNS

To prepare CDNS**,** the melting method was used. Briefly, β-CD (1 mmol) as monomer was added to the melted cross-linking agent, diphenyl carbonate (8 mmol). The polymerization reaction proceeded under stirring at 120 °C under atmospheric condition for 10 h. At the end of the reaction, the obtained white solid was cooled to room temperature and crushed to fine powder. Next, CDNS purification was achieved by addition of an aqueous NaOH solution (1 M) to an aqueous suspension of CDNS in order to let the phenol by-product become soluble as sodium phenoxide. The CDNS was then filtered off, washed with acetone, and distilled water. Further purification was carried out by Soxhlet extraction with EtOH for 4 h. The resulting CDNS was then dried in the oven at 90 °C for 10 h.

#### 3.2.2. Synthesis of Amine-Functionalized CDNS (CDNS-N)

APTES solution (4 mL in 20 mL of dry toluene) was added in a dropwise manner to a stirring suspension of CDNS (1.2 g) in dry toluene (40 mL). The obtained mixture was then irradiated with ultrasonic irradiation at a power of 100 W for 30 min. The resulting suspension was subsequently refluxed at 110 °C under nitrogen atmosphere for 24 h. After completion of reaction, the white solid was filtered off and washed with dry toluene repeatedly and then dried at 80 °C overnight to afford 1.3 g CDNS-N.

#### 3.2.3. Growing Polymer on CDNS (CDNS-N/PMelamine)

CDNS-N (1.0 g) was dispersed in 20 mL dry THF in a clean round-bottom flask and then sonicated for 20 min. Subsequently, TCT (2.0 g, 10 mmol) was added to the suspension and afterwards, 2.0 g (32 mmol) of EDA was slowly dropped into the stirring mixture. The resulting mixture was then kept at 0 °C. After the addition of 2.0 g (14 mmol) of K_2_CO_3_, the resulting mixture was stirred under atmospheric condition for 4 h at room temperature and then refluxed at 50 °C for 24 h. Upon completion of the polymerization reaction, the obtained solid support (denoted as CDNS-N/PMelamine) was filtered and washed three times with methanol and then dried at 60 °C for 12 h to afford 1.28 g CDNS-N/PMelamine.

#### 3.2.4. Preparations of Cuscuta Epithymum Extract

Fresh leaves of *Cuscuta epithymum* were collected from Banaruiyeh District, in Larestan, Iran. First, the collected *Cuscuta epithymum* (2 g) were crushed in porcelain mortar. Then, the resulting powder was mixed thoroughly with 100 mL deionized water (DW) and boiled for 60 min at 80 °C. The extract was then obtained by cooling the mixture and simple filtration.

#### 3.2.5. Synthesis of Ag NPs and Their Embedding into CDNS-N/PMelamine: Synthesis of Ag@CDNS-N/PMelamine

**Figure 11:**
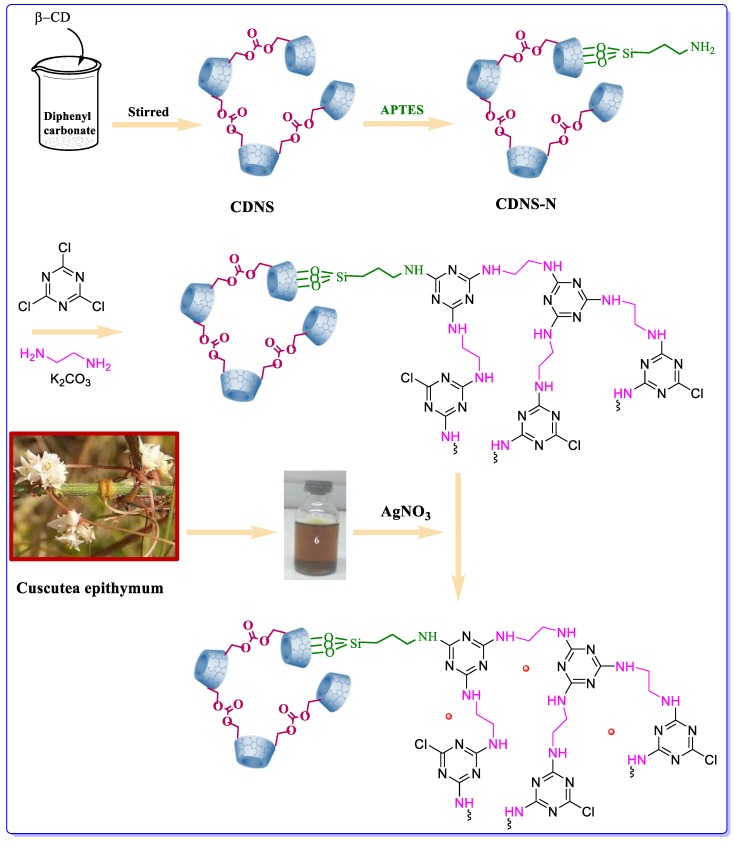
The schematic procedure of the synthesis of the proposed structures.

CDNS-N/PMelamine (1 g) was dispersed into a solution containing 0.1 g of AgNO_3_ in 20 mL DW and kept under stirring at room temperature under atmospheric condition for 30 min. The adsorption of Ag^+^ ions on the surfaces of CDNS-N/PMelamine was conducted using electrostatic attraction. Then, the fresh extract (2 mL in 20 mL DW) as a reducing agent was added immediately to the suspension. Upon addition of the bio-based reducing agent, the solution turned black, confirming the reduction of Ag^+^ to metallic silver (Ag). The mixture was then stirred continuously under atmospheric condition for 12 h to immobilize Ag NPs into the CDNS-N/PMelamine and forming Ag@CDNS-N/PMelamine. Finally, the resulting product was separated and repeatedly washed using EtOH/DW and then dried in electronic oven at 60 °C for 12 h to obtain 0.9 g catalyst. Figure 11 presents a schematic illustration of synthesis of the proposed structures. Notably, to prepare the control catalysts, Ag@CDNS and Ag@PMelamine, the same protocol was applied, except CDNS and PMelamine were used as supports respectively.

### 3.3. General Procedure for the Synthesis Xanthenes Derivative

In a typical procedure, to a mixture of aldehyde (1 mmol) and dimedone (2 mmol) in 1:2 H_2_O:EtOH (3 mL), Ag@CDNS-N/PMelamine (0.03 g) was added and the mixture was stirred under atmospheric condition at 50 °C for 3 h. The reaction progress was monitored by TLC and at the end of the reaction, EtOH (20 mL) was added to the reaction mixture and the catalyst was separated via simple filtration. To purify the organic product, it was recrystallized from EtOH.

## 4. Conclusions

A novel covalent hybrid system composed of CDNS and multi-nitrogen atom-containing polymer were prepared through simple procedure, including functionalization of CDNS followed by its reaction with ethylenediamine and 2,4,6-trichloro-1,3,5-triazine. The resulting compound was then applied as a catalyst support for the immobilization of Ag(0) nanoparticles, reduced by *Cuscuta epithymum* extract as a naturally derived reducing agent. The catalyst, Ag@CDNS-N/PMelamine, was successfully applied for promoting the two component reaction of aldehydes and dimedone for the formation of xanthenes in aqueous media under mild reaction condition. The catalytic activity of the catalyst was superior to Ag@CDNS and Ag@PMelamine and showed high recyclability with low Ag leaching. The high catalytic performance of the catalyst was attributed to the capability of CD for the formation of inclusion complex with hydrophobic substrates and its role as a phase transferring agent in bringing the substrates in close contact with the catalytic sites. Moreover, CD could act as capping agent for Ag NPs. On the other hand, the multi-nitrogen functionalities on the polymer backbone could improve anchoring of Ag NPs and suppress their leaching.

## Figures and Tables

**Table 1:** The quantitative results of EDS analysis.

Element	Weight (%)	Atomic (%)
C	44.13	51.87
N	39.42	39.74
O	6.55	5.78
Si	0.82	0.41
Cl	3.04	1.21
Ag	5.19	0.68
K	0.86	0.31

**Table 2:** Optimization of reaction condition for the synthesis of model xanthene.

Entry	Solvent	Temp. (°C)	Catalyst Amount (g)	Yield (%)
1	H_2_O	25	0.02	76
2	EtOH	25	0.02	79
3	H_2_O:EtOH (1:2)	25	0.02	80
4	THF	25	0.02	70
5	CH_3_CN	25	0.02	72
6	H_2_O:EtOH (1:2)	50	0.02	85
7	H_2_O:EtOH (1:2)	70	0.02	85
8	H_2_O:EtOH (1:2)	50	0.03	92
9	H_2_O:EtOH (1:2)	50	0.04	92

**Table 3:** Comparison of the catalytic activity of Ag@CDNS-N/PMelamine with some control catalysts.

Entry	Catalyst	Yield at Optimum Reaction Condition of the Catalyst (%) ^a^	Yield at Optimum Reaction Condition of Each Control Sample (%)
1	Ag@CDNS	70	78 ^b^
2	Ag@CDNS-N	70	78 ^b^
3	Ag@PMelamine	72	80 ^c^
4	Ag@CDNS-N/PMelamine	92	92 ^a^

^a^ 0.03 g of catalyst at 50 °C in the mixture of H_2_O:EtOH. ^b^ use of 0.04 g of Ag@CDNS at 60 °C in the mixture of H_2_O:EtOH. ^c^ 0.035 g of Ag@PMelamine at 50 °C in the mixture of H_2_O:EtOH.

**Table 4:**
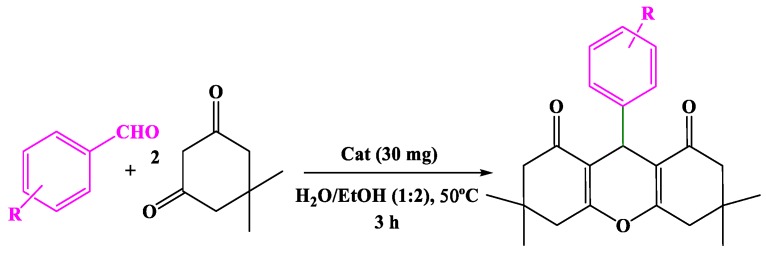
Synthesis of various xanthenes under Ag@CDNS-N/PMelamine catalysis.

Entry	Substrate	Yield (%) ^a^
1	Benzaldehyde	92
2	4-NO_2_-benzaldehyde	95
3	2-NO_2_-benzaldehyde	93
4	4-Me-benzaldehyde	98
5	4-MeO-benzaldehyde	95
6	2-MeO-benzaldehyde	90
7	4-Cl-benzaldehyde	95
8	Furfural ^b^	90

^a^ Isolated Yield. ^b^ 91% in 4 h (65% in 3 h).

**Table 5:** The comparison of the catalytic activity of the catalyst with some other reports for the synthesis of 3,4,6,7-Tetrahydro-3,3,6,6-tetramethyl-9-phenyl-2*H*-xanthene1,8-(5*H*,9*H*)-dione. ^a^

Entry	Catalyst	Solvent	Time h:min	Temp. (°C)	Quantity	Yield (%)	Ref.
1	Ag@CDNS-N/PMelamine	H_2_O:EtOH	03:00	50	0.03 g	92	-
2	Fe_3_O_4_@SiO_2_–SO_3_H	-	00:4	110	0.05 g	97	[33]
3	Silica-bonded S-sulfonic acid (SBSSA)	EtOH	10:00	Reflux	0.03 g	98	[34]
4	Nano-ZnO	-	02:00	100	10 mol%	Trace	[32]
5	Barium Perchlorate	EtOH	03:00	Reflux	15 mol%	95	[47]
6	Nano titania-supported sulfonic acid (n-TSA)	-	01:10	90	0.013 g	91	[32]
7	Nano-NiO	-	02:00	100	10 mol%	Trace	[32]
8	Fe_2_(SO_4_)_3_.7H_2_O	-	01:30	120	10 mol%	86	[48]

^a^ The catalyst quantity was measured for 1 mmol benzaldehyde.

## Supplementary Materials

The following are available online, Figure S1. SEM image, EDS analysis and Elemental mapping analysis of CDNS, Figure S2. SEM image, EDS analysis and Elemental mapping analysis of CDNS-N, Figure S3. SEM image, EDS analysis and Elemental mapping analysis of CDNS-N/PMelamine.
