# Supplementary material for: Ag Nanoparticles Stabilized on Cyclodextrin Polymer Decorated with Multi-Nitrogen Atom Containing Polymer: An Efficient Catalyst for the Synthesis of Xanthenes
Source: Molecules. 2020 Jan 7;25(2):241. doi: 10.3390/molecules25020241 (PMC7024309; doi:10.3390/molecules25020241)
Supplement: Supplementary file 1 [file molecules-25-00241-s001.pdf]

Supporting information

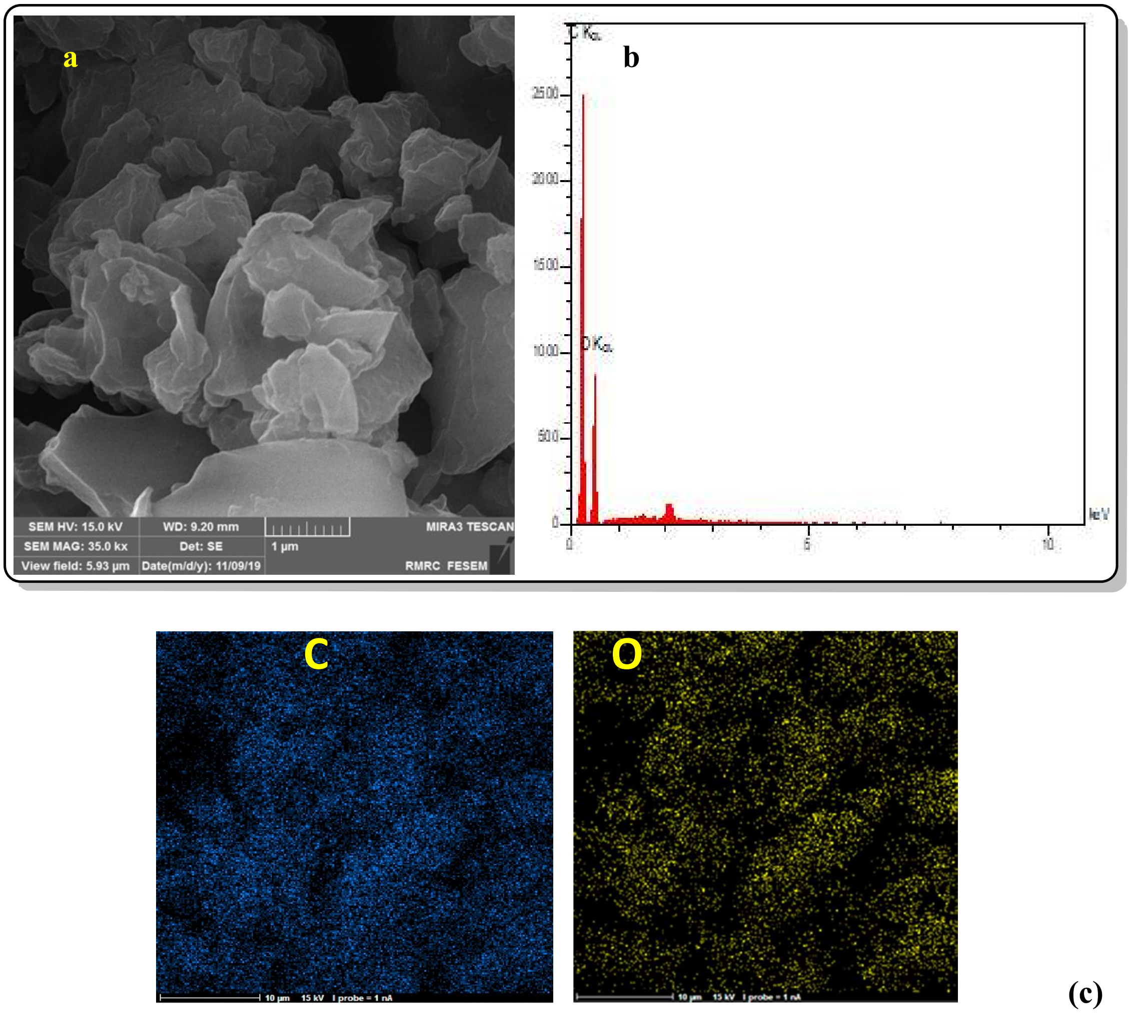

Figure S1. SEM image (**a**), EDS analysis (**b**) and Elemental mapping analysis (**c**) of CDNS.

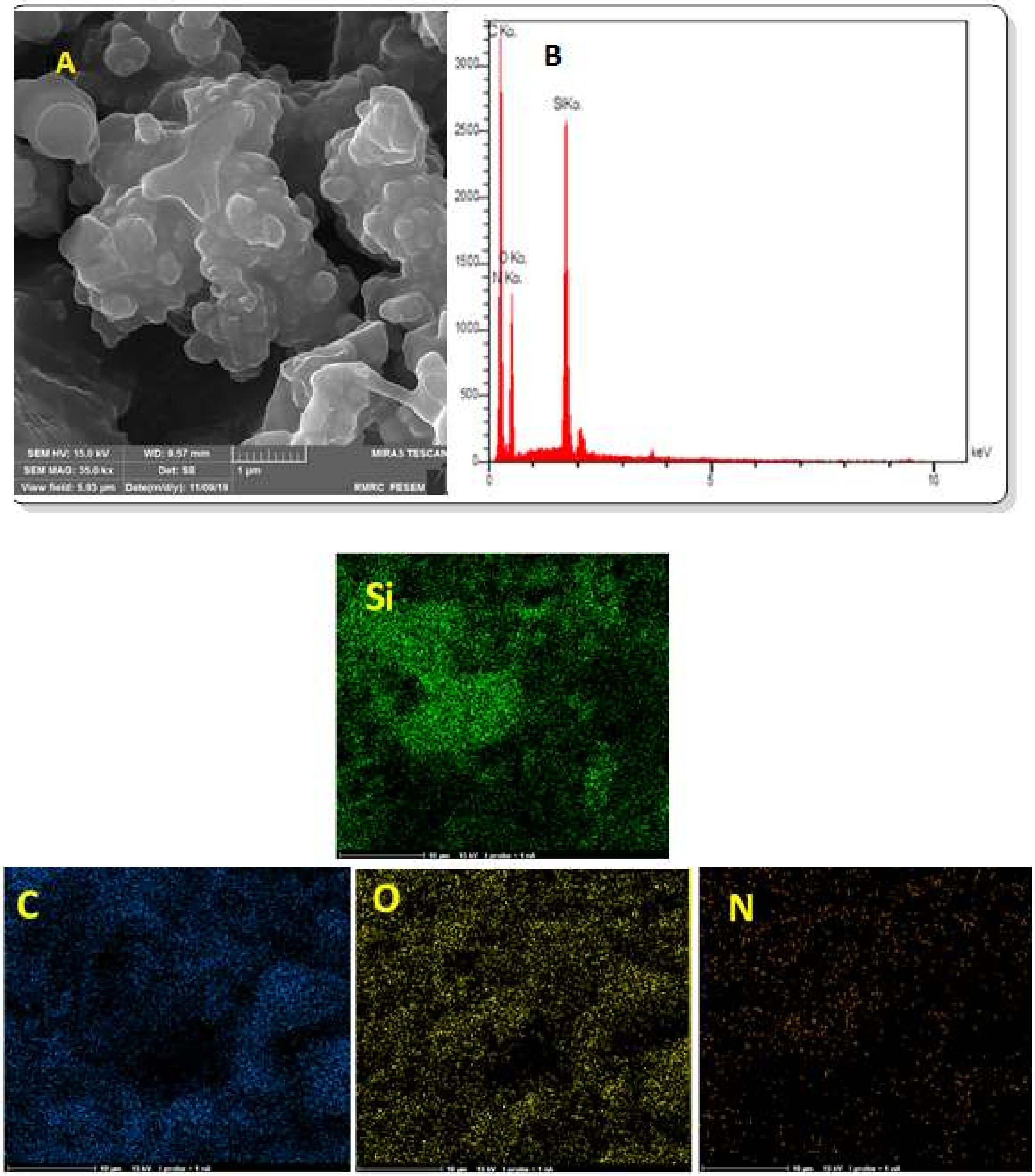

C

Figure S2. SEM image (**a**), EDS analysis (**b**) and Elemental mapping analysis (**c**) of CDNS-N.

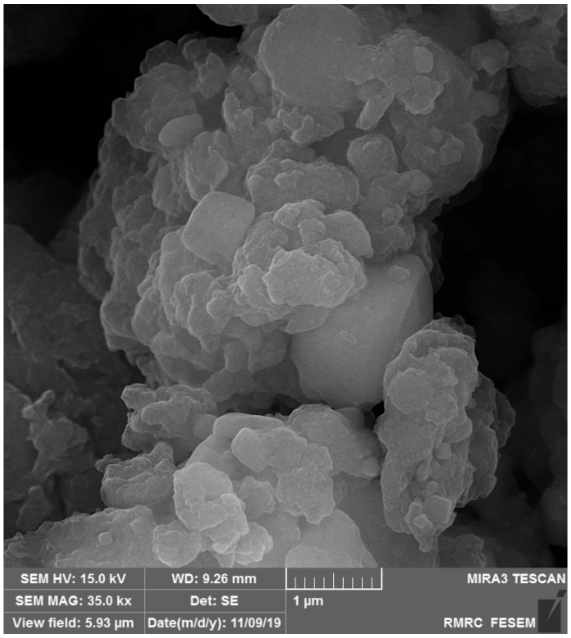

SEM HV: 15.0 kV
WD: 9.26 mm
MIRA3 TESCAN
SEM MAG: 35.0 kx
Det: SE
1 μm
View field: 5.93 μm
Date(m/d/y): 11/09/19
RMRC FESEM

A

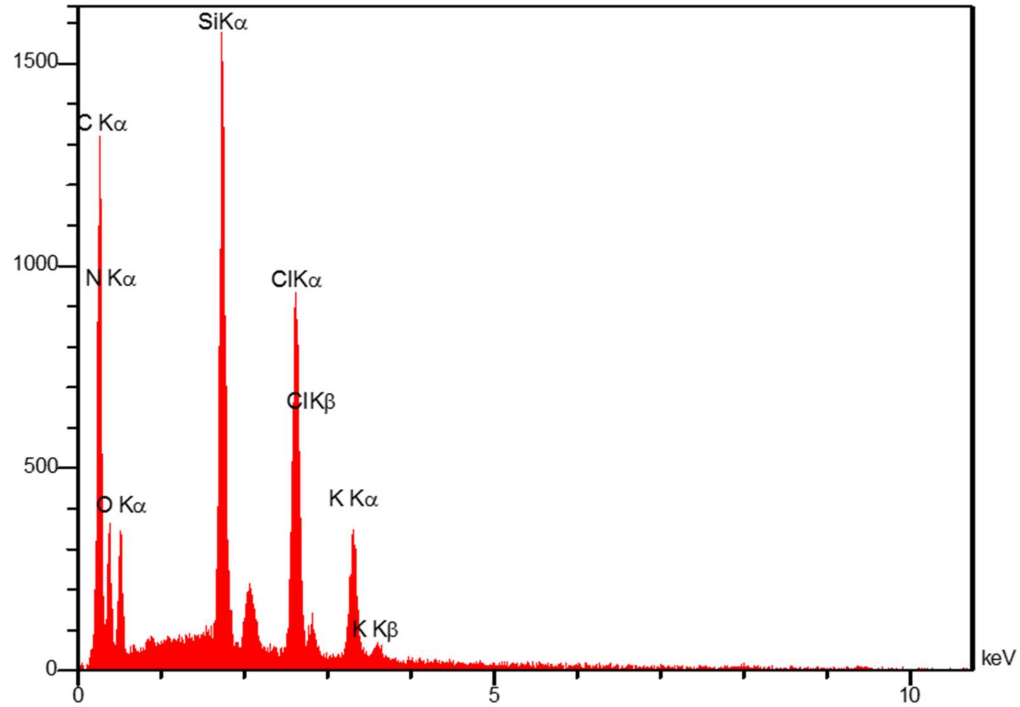

SiKα
C Kα
N Kα
ClKα
ClKβ
O Kα
K Kα
K Kβ
1500
1000
500
0
5
10
keV

B

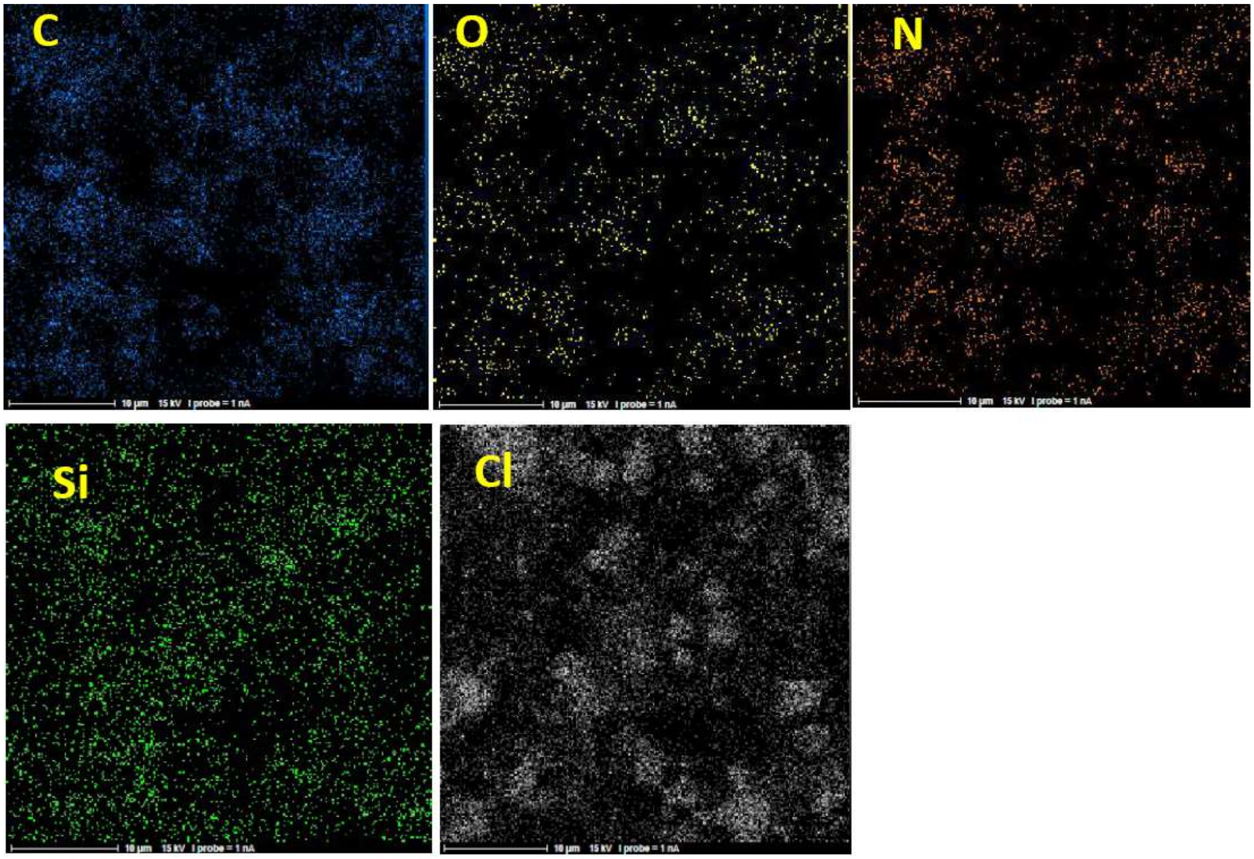

Figure S3. SEM image (A), and Elemental mapping analysis (B) of CDNS-N/PMelamine
